# Correction: Li et al. Recovery Behavior of the Macro-Cracks in Elevated Temperature-Damaged Concrete after Post-Fire Curing. *Materials* 2022, *15*, 5673

**DOI:** 10.3390/ma17071563

**Published:** 2024-03-29

**Authors:** Lang Li, Yao Chen, Chao He, Chong Wang, Hong Zhang, Qingyuan Wang, Yongjie Liu, Guomin Zhang

**Affiliations:** 1 Failure Mechanics & Engineering Disaster Prevention, Key Laboratory of Sichuan Province, College of Architecture & Environment, Sichuan University, Chengdu 610065, China; yaochen@scu.edu.cn (Y.C.); hechao@scu.edu.cn (C.H.); wangchongscu@163.com (C.W.); zzhanghong@scu.edu.cn (H.Z.); wangqy@scu.edu.cn (Q.W.); 2 Key Laboratory of Deep Underground Science and Engineering, Ministry of Education, Sichuan University, Chengdu 610065, China; 3 Civil and Infrastructure Engineering Discipline, School of Engineering, RMIT University, Melbourne, VIC 3001, Australia; kevin.zhang@rmit.edu.au

Following publication, concerns relating to the relevance of a number of citations recommend by a peer reviewer were brought to the attention of the Editorial Office.

Adhering to our complaints procedure, an investigation was conducted by the Editorial Office and Editorial Board, which confirmed that the references [3–5] lack sufficient relevance to this publication [1], and the recommendation of these citations by the reviewer was considered to breach MDPI’s reviewer policy (https://www.mdpi.com/reviewers#_bookmark11). As a result, the authors, the Editorial Office and Editorial Board have decided to remove the references [3–5] in Section 1 as per MDPI’s correction policy (https://www.mdpi.com/ethics#_bookmark30). References [6–32] in the original publication were updated to [3–29] accordingly.

The authors state that the scientific conclusions are unaffected. This correction was approved by the Academic Editor. The original publication has also been updated.

## References

[1] Li L., Chen Y., He C., Wang C., Zhang H., Wang Q., Liu Y., Zhang G. (2022). Recovery Behavior of the Macro-Cracks in Elevated Temperature-Damaged Concrete after Post-Fire Curing. Materials.

